# Single Anastomosis Duodeno-ileostomy (SADI-S) Versus One Anastomosis Gastric Bypass (OAGB-MGB) as Revisional Procedures for Patients with Weight Recidivism After Sleeve Gastrectomy: a Comparative Analysis of Efficacy and Outcomes

**DOI:** 10.1007/s11695-020-04933-2

**Published:** 2020-08-26

**Authors:** Moataz Bashah, Ammar Aleter, Jawher Baazaoui, Ayman El-Menyar, Antonio Torres, Asaad Salama

**Affiliations:** 1 Department of Bariatric and Metabolic Surgery, Hamad General Hospital, Doha, Qatar; 2 Department of Surgery, Weill Cornell Medical College, Doha, Qatar; 3 Clinical Research, Trauma and Vascular Surgery Section, Hamad General Hospital, PO Box 3050, Doha, Qatar; 4 Clinical Medicine, Weill Cornell Medical College, Doha, Qatar; 5 Department of Surgery, Hospital Clinico San Carlos, Complutense University of Madrid, Madrid, Spain

**Keywords:** Revisional surgery, Sleeve gastrectomy, Single anastomosis duodeno-ileostomy, One anastomosis gastric bypass

## Abstract

**Purpose:**

Many revisional procedures are available for unsuccessful laparoscopic sleeve gastrectomy (LSG) in patients with complications or weight recidivism. Single anastomosis duodeno-ileal bypass (SADI-S) and one anastomosis gastric bypass (OAGB-MGB) are two revisional procedures to address the problem of weight recidivism. We aimed to evaluate the efficacy and outcomes of the 2 revisional approaches (SADI-S vs. OAGB-MGB).

**Materials and Methods:**

A retrospective analysis of prospectively collected database of patients who underwent SADI-S or OAGB-MGB as a revisional procedure for weight recidivism after primary LSG with a minimum 1-year follow-up. Weight loss, comorbidities, nutritional deficiencies, complications, and outcomes were compared in the 2 procedures.

**Results:**

Ninety-one patients were included in the study (42 SADI-S and 49 OAGB-MGB). There was a significant weight loss (total weight loss percentage, TWL%) at 1-year follow-up observed for SADI-S when compared to OAGB-MGB (23.7 ± 5.7 vs. 18.7 ± 8.5, *p* = 0.02). However, this difference was not statistically significant at 18 months (26.4 ± 7.3 vs. 21.2 ± 11.0, *p* = 0.25). Remission of comorbidities (diabetes mellitus and hypertension) was comparable. Although OAGB-MGB had higher complication rate than SADI-S, the difference was not statistically significant (*p* = 0.39). No mortality was reported in the study groups.

**Conclusion:**

Both SADI-S and OAGB-MGB are effective and safe revisional procedures for weight regain after LSG. The short-term outcomes are comparable; however, SADI-S is associated with less upper gastrointestinal complications and could be a better option for patients suffering from GERD post-LSG. Moreover, the underlying bile reflux may get worse with OAGB-MGB. However, further prospective larger studies are needed.

**Electronic supplementary material:**

The online version of this article (10.1007/s11695-020-04933-2) contains supplementary material, which is available to authorized users.

## Introduction

Over the past decade, bariatric surgery has become the main approach to deal with obesity and its related comorbidities, showing great efficacy and durability in managing this epidemic disease [1, 2]. However, it has been noticed that significant percentage of patients regain an excess weight after bariatric surgery, especially after laparoscopic sleeve gastrectomy (LSG) and mainly in patients with a higher body mass index (BMI) before the primary procedure [3, 4]. Therefore, such patients would not sufficiently benefit from a stand-alone LSG and are advised to undergo a revisional surgery. For this reason, the number of patients undergoing revisional/secondary bariatric surgeries has increased significantly [5, 6]. This growing number of patients stimulates the necessity to investigate the effectiveness of these revisional procedures which make them one of the main bariatric research topics recently.

Several surgical procedures are currently used as a revisional procedure for weight regain post LSG. Single anastomosis duodeno-ileostomy surgery (SADI-S) is one of the most recently developed procedures for revision. It is considered a technically less challenging procedure than the classic duodenal switch (DS) with simpler technique, reduced number of anastomosis and apparently has a similar outcome in terms of weight loss [7, 8]. Earlier researches of SADI-S as a primary procedure, with 3-year follow-up period showed almost 100% excess weight loss [7, 9]. Another surgical procedure proposed as an effective option for revision is the one anastomosis gastric bypass/mini gastric bypass (OAGB-MGB); it has been reported as a well-tolerated and potent revisional option in the long term [10]. However, studies comparing SADI-S and OAGB-MGB as revisional procedures after unsuccessful LSG are scarce [11]. In this study, we report short-to-medium term (1-year minimum) experience comparing SADI-S and OAGB-MGB as revisional procedures after weight recidivism post-LSG, focusing mainly on the operative management and postoperative outcomes.

## Methods

This is a retrospective observational study of a prospectively collected database, based on data collected from the electronic medical records. All patients who underwent SADI-S or OAGB-MGB as a revisional procedure for weight recidivism post-LSG in the duration between 1st January 2016 and 1st August 2017 at Hamad General Hospital, Qatar, were included. Patients who underwent SADI-S or OAGB-MGB as a primary procedure with insufficient follow-up (< 1 year) were excluded.

The primary outcome of the study was the loss of weight over 1-year period. Loss of weight was estimated using multiple parameters including BMI, excess weight loss percentage (EWL%), and total weight loss percentage (TWL%). In this study, EWL% and TWL% were calculated with the weight before SADI or OAGB-MGB as a baseline.

Secondary parameters evaluated before and after 1 year of surgery included metabolic profile data, specific obesity-related diseases such as diabetes mellitus type 2 (T2D), hypertension, and gastroesophageal reflux disease (GERD) along with the evaluation of various blood markers and postoperative complications. Remission of T2D was defined as hemoglobin A1c (A1C) < 6.5 and/or free blood glucose < 100 mg/dl, and remission of hypertension was defined as normotensive with blood pressure < 130/90 mmHg off medications [10]. GERD was diagnosed preoperatively based on patient symptoms, dependency on medications, preoperative oesophago-gastro-duodenoscopy (OGD), and upper gastrointestinal series.

We used a symptom’s score to assess the resolution of GERD in which complete resolution was considered when symptoms were absent and no medication used, and improvement was considered with improved symptom severity, frequency, or decreased medication use [12]. Blood markers evaluated pre- and post-procedures included hemoglobin (Hb), total serum protein level, serum calcium, triglycerides, cholesterol, LDL, HDL, albumin, ferritin, iron, zinc, INR, vitamin B12, and vitamin D. The postoperative complications were bleeding, anastomosis leakage, anastomotic ulcer, abdominal collection, internal herniation, vitamin B12, and vitamin D deficiencies, and defecation complaints (either obstipation; no defecation for at least 3 days or steatorrhea).

### Surgical Procedures

#### SADI-S

All patients had a prior LSG. None of the patients underwent dissection of the sleeved stomach or re-sleeve. Retroduodenal dissection was carried out with an ultrasonic energy device distal to the gastroduodenal artery. The peritoneum was divided lateral to the 2nd part of the duodenum and distal to the common bile duct. The peritoneal covering the hepatoduodenal ligament was opened just at the level of the superior duodenal wall border. The first part of the duodenum was divided about 2–4 cm distal to the pyloric ring with a linear stapler 60–3.5 mm. Ileocecal junction was identified, 250–300-cm ileum was measured and brought up to the proximally divided duodenum and sutured to the staple line with running PDS 3.0. Openings were made on the pouch and ileal loop and “end-to-side” two-layer duodeno-ileostomy was created using a hand-sewn anastomosis technique with 3/0 barbed suture. Methylene blue test was routinely performed to check the anastomosis, and drains were not used routinely.

#### OAGB-MGB

All patients had a prior LSG. The gastric sleeve was usually transected at the level of the incisura angularis to create the new gastric pouch over a 36-Fr tube using a linear stapler type along the tube to the angle of His (trimming the previous gastric sleeve). The duodenojejunal junction was identified and a loop of 150 to 200 cm was measured and ascended antecolic to create a side-to-side gastro-jejunal anastomosis using a linear stapler and V-loc stitches. A methylene blue test for leak was always performed. Drains were not used in any case.

All patients were given the same postoperative instructions including a liquid diet (with protein supplements) for the first 2 weeks, proton pump inhibitors (PPIs) for 3 months. For both procedures, all patients were routinely maintained on multivitamins postoperatively. Other supplements, minerals, trace elements were given selectively as needed based on routine postoperative follow-up blood tests.

#### Statistical Analysis

Data were presented as proportions or mean ± standard deviation (SD). Normality of distribution for continuous variables was checked. Independent sample *t* test (parametric test) was used for normally distributed variables.

Chi-square test was used to compare proportions (categorical variables) between the groups. Means of continuous variables across the study groups were compared using *t* test and the differences were considered as significant at 2-tailed *p* value level < 0.05. Data analysis was carried out using the Statistical Package for Social Sciences version 21 (SPSS Inc., Chicago, IL).

Ethical approval for this study was obtained from the Institutional Review Board at Hamad Medical Corporation, Medical Research center, Doha, Qatar (MRC-01-19-335). This study follows the STROBE checklist (Suppl Table).

## Results

### Preoperative Characteristics

A total of 91 patients were included in the study; 42 patients underwent SADI-S and 49 underwent OAGB-MGB as revisional procedures for weight regain post LSG and had a follow-up for at least 12 months. The mean age in both study groups was approximately 38 years with a higher proportion of females. The mean pre-LSG weight was 133 ± 28 in the OAGB-MGB and 139 ± 27 in the SADI-S group. The mean pre-LSG BMI was 52 ± 11 for OAGB-MGB and 50 ± 8 kg/m^2^ for the SADI-S group.

### OAGB-MGB

Table 1 describes the demographics, anthropometrics, and complications of patients who underwent OAGB-MGB. Male to female ratio was 1:6 with a mean age of 38 ± 9 years. The mean preoperative BMI was 43.6 ± 7.4 kg/m^2^ that was dropped to 35.3 ± 6.5 1 year post-revisional surgery. Table 2 shows a comparison of blood marker levels before and after the procedure. There was a significant improvement in the laboratory tests in terms of the A1C, serum cholesterol, HDL, and LDL. OAGB-MGB procedure was found to have a drop in Hb after 1 year (*p* = 0.006).

**Table 1:** Demographic, anthropometric, complications, and outcome data of OAGB-MGB patients

Gender (male:female)	1: 6
Age (mean ± standard deviation)	37.83 ± 9.36
No. of years follow-up	3.8 ± 1.4
Weight (kg)	
Before LSG Before revisional procedure 1 year after revision	133 ± 27.8 114.2 ± 21.1 92.2 ± 16.5 (*p* value 0.0001)
BMI	
Before LSG Before revisional procedure 1 year after revision	52.32 ± 11.43 43.6 ± 7.4 35.3 ± 6.5 (*p* value 0.0001)
TWL% post-revisional procedure	
At 1 year At 18 months	21.6 ± 8.9 21.2 ± 11.0 (*p* value 0.69)
EWL% post-revisional procedure	
At 1 year At 18 months	53.1 ± 22.6 52.1 ± 27.9 (*p* value 0.68)
Postoperative complications (*n*)	
Staple line leak Anastomotic ulcer Bile reflux De novo GERD Nutritional deficiency Revisional surgery Mortality	1 (converted to RYGB) 3 3 (1 converted to RYGB) 3 1 2 (converted to SADI-S) 0

*OAGB-MGB* one anastomosis gastric bypass, *LSG* laparoscopic sleeve gastrectomy, *BMI* body mass index, *TWL%* total weight loss percentage, *EWL%* excess weight loss percentage, *GERD* gastroesophageal reflux disease, *RYGB* Roux-en-Y gastric bypass, *SADI* single anastomosis duodeno-ileostomy

**Table 2:** Comparison of blood markers level before and after OAGB-MGB as revisional procedure

Parameters	Before OAGB-MGB (mean ± SD)	12 months after OAGB-MGB (mean ± SD)	*p* value
Hemoglobin A1C	5.45 ± 0.69	5.1 ± 0.6	0.0001*
Serum protein	69.43 ± 4.40	68.1 ± 4.9	0.06
Hemoglobin level	12.07 ± 1.52	11.7 ± 1.3	0.006*
Serum albumin	36.42 ± 3.15	36.6 ± 4.4	0.81
Serum zinc	12.25 ± 1.49	11.3 ± 1.7	0.15
Serum vitamin B12	255.05 ± 87.56	291.1 ± 146.9	0.18
International normalized ratio (INR)	1 ± 0.04	0.9 ± 0.04	0.49
Serum vitamin D	17.80 ± 9.95	19.2 ± 11.7	0.31
Triglycerides level	1.16 ± 0.56	0.9 ± 0.3	0.08
Serum cholesterol level	5.09 ± 1	4.4 ± 0.9	0.0001*
High-density lipoprotein (HDL)	1.42 ± 0.42	1.5 ± 0.4	0.04
Low-density lipoprotein (LDL)	3.13 ± 0.91	2.6 ± 0.7	0.0001*
Serum iron	12.91 ± 6.19	11.3 ± 5.7	0.19
Aspartate aminotransferase (AST)	16.81 ± 6.21	18.8 ± 5.9	0.07
Alanine aminotransferase (ALT)	17.50 ± 9.12	17.8 ± 6.6	0.76
Serum bilirubin	9.42 ± 6.89	7.9 ± 3.5	0.11

*SD* standard deviation. *High significant

### SADI-S

Table 3 shows demographics, anthropometrics, and complications in patients who underwent SADI-S. The male to female ratio was 2:5. There was a significant drop in BMI after 1 year of SADI-S (from 43.7 ± 7.1 to 34.3 ± 6.1). Also, the TWL% and EWL% were significantly increased at 6 and 12 months. Table 4 provides a comparative analysis of blood marker levels before and after the SADI-S procedure. There were significant increases in the serum vitamin B12 and iron; however, there were significant decreases in the A1c, vitamin D, triglyceride, HDL, and LDL.

**Table 3:** Demographic, anthropometric, complications, and outcome data of patients who underwent SADI-S as revisional procedure

Gender (male:female)	2: 5
Age (mean ± standard deviation)	38.0 ± 9.0
BMI	
Before LSG Before revisional procedure 1 year after revision	50.43 ± 8.54 43.7 ± 7.1 34.3 ± 6.1 (*p* value < 0.0001)
TWL% post-revisional procedure	
At 6 months At 12 months	16.4 ± 4.76 20.8 ± 6.0 (*p* value < 0.001)
EWL% post-revisional procedure	
At 6 months At 12 months	40.9 ± 15.9 51.3 ± 18.8 (*p* value < 0.001)
Postoperative complications (*n*)	
Abdominal collection Steatorrhea Nutritional deficiency Mortality	1 6 1 0

*SADI-S* single anastomosis duodeno-ileostomy, *LSG* laparoscopic sleeve gastrectomy, *BMI* body mass index, *TWL%* total weight loss percentage, *EWL%* excess weight loss percentage

**Table 4:** Comparison of blood markers levels before and after SADI as revisional procedure for weight regain

Blood markers	Before SADI-S (mean ± SD)	12 months after SADI-S (mean ± SD)	*p* value
Hemoglobin A1C	6.03 ± 1.23	5.11 ± 0.57	0.042
Total serum protein	69.19 ± 4.76	64.47 ± 8.82	0.480
Hemoglobin value	12.63 ± 1.69	13.89 ± 12.51	0.618
Serum albumin	35.54 ± 3.49	36.57 ± 9.40	0.471
Serum zinc	11.80 ± 2.64	9.02 ± 0.90	0.329
Serum vitamin B12	303.81 ± 302.17	353.45 ± 305.25	0.010
International normalized ratio (INR)	1 ± 0.06	1.05 ± 0.06	0.339
Serum vitamin D	15.42 ± 5.75	11.76 ± 5.32	0.006*
Serum triglycerides	1.13 ± 0.34	0.83 ± 0.23	0.005*
Serum cholesterol	5.24 ± 0.98	4.12 ± 0.91	0.286
High-density lipoprotein (HDL)	1.43 ± 0.26	1.20 ± 0.27	0.001*
Low-density lipoprotein (LDL)	3.30 ± 0.91	2.38 ± 0.69	0.041
Serum iron	10.01 ± 5.17	11.69 ± 5.69	0.045

*SD* standard deviation. *High significant

### SADI-S Versus OAGB-MGB

Table 5 shows the results of a comparative analysis between the 2 procedures. The mean number of years between the initial LSG and the revisional procedure was 24 to 48 months for both procedures. Preoperative weight and BMI across both groups were comparable. The TWL% after 12 months was significantly higher with SADI-S in comparison to OAGB-MGB; otherwise, the other anthropometric parameters were comparable. There was no significant difference in TWL% between the procedures after 18 months. In addition, EWL% after 1 year or 18 months were comparable between the 2 procedures (*p* > 0.05).

**Table 5:** Comparative analysis of anthropometric measures before and after revisional surgeries

Parameters	OAGB-MGB (mean ± SD)	SADI-S (mean ± SD)	*p* value
BMI before LSG	52.32 ± 11.43	50.43 ± 8.54	0.364
Pre-revisional BMI	43.58 ± 7.38	43.90 ± 7.37	0.832
TWL% (in relation to pre-LSG value)	30.83 ± 9.41	29.17 ± 13.09	0.543
EWL% (in relation to pre-LSG value)	59.20 ± 18.53	59.12 ± 27.23	0.987
12 months BMI	35.32 ± 6.47	34.09 ± 5.74	0.460
12 months TWL%	18.68 ± 8.52	23.70 ± 5.72	0.019
12 months EWL%	47.10 ± 21.95	57.63 ± 19.85	0.068
18 months BMI	34.68 ± 7.19	34.91 ± 11.22	0.948
18 months TWL%	21.19 ± 11.01	26.40 ± 7.29	0.250
18 months EWL%	52.13 ± 27.89	65.78 ± 31.62	0.274

*OABG* one anastomosis gastric bypass, *SADI* single anastomosis duodeno-ileostomy, *LSG* laparoscopic sleeve gastrectomy, *SD* standard deviation, *BMI* body mass index, *EW* excess weight than expected normal, *WL* weight loss, *TWL%* total weight loss percentage, *EWL%* excess weight loss percentage

Table 6 shows the results of a comparative analysis of blood markers between the 2 procedures. At 1 year, there were significantly higher levels of serum protein, serum zinc, serum vitamin D, and HDL in patients who had OAGB-MGB compared to SADI-S. The pre- and postoperative A1C values were comparable between the 2 procedures.

**Table 6:** Comparative analysis of blood markers levels before and after revisional surgeries

Parameters	OAGB-MGB (mean ± SD)	SADI-S (mean ± SD)	*p* value
Preoperative hemoglobin A1C	5.48 ± 0.68	5.77 ± 1.06	0.151
12 months hemoglobin A1C	5.11 ± 0.57	5.11 ± 0.52	0.998
Preoperative protein level	69.43 ± 4.40	68.71 ± 5.38	0.521
12 months protein level	68.06 ± 4.97	64.83 ± 8.33	0.046
Preoperative hemoglobin	12.07 ± 1.50	12.63 ± 1.69	0.100
12 months hemoglobin	11.67 ± 1.27	13.89 ± 12.37	0.229
Preoperative serum albumin	36.14 ± 3.12	35.54 ± 3.49	0.216
12 months serum albumin	36.59 ± 4.44	36.54 ± 9.20	0.973
Preoperative serum zinc	12.14 ± 1.80	12.15 ± 2.06	0.998
12 months serum zinc	11.24 ± 1.69	9.16 ± 1.80	0.004*
Preoperative serum vitamin B12	248.28 ± 84.54	310.51 ± 267.72	0.160
12 months vitamin B12	282.29 ± 144.32	395.76 ± 357.72	0.085
Preoperative INR	1 ± 0.04	1 ± 0.07	0.857
12 months INR	0.9 ± 0.04	1.02 ± 0.07	0.135
Preoperative vitamin D level	17.46 ± 10.24	13.84 ± 5.97	0.078
12 months vitamin D level	18.97 ± 11.58	12.19 ± 4.98	0.007*
Preoperative serum triglycerides	1.16 ± 0.54	1.13 ± 0.33	0.833
12 months serum triglycerides	0.97 ± 0.34	0.83 ± 0.21	0.146
Preoperative serum cholesterol	5.13 ± 1.01	5.18 ± .86	0.818
12 months serum cholesterol	4.41 ± 0.97	4.17 ± 0.81	0.333
Preoperative HDL level	1.45 ± 0.49	1.43 ± 0.25	0.831
12 months HDL level	1.52 ± 0.44	1.19 ± 0.24	0.002*
Preoperative LDL level	3.18 ± 0.94	3.24 ± 0.76	0.801
12 months LDL level	2.52 ± 0.73	2.44 ± 0.63	0.671
Preoperative serum iron	12.46 ± 6.02	10.77 ± 5.95	0.229
12 months serum iron	11.64 ± 5.89	11.66 ± 5.69	0.991

*Hb* hemoglobin, *INR* international normalized ratio, *HDL* high-density lipoprotein, *LDL* low-density lipoprotein. *High significant

### Nutritional Deficiency

When comparing both procedures in terms of nutritional deficiency (Hb, albumin, vitamin B12, and INR), no statistically significant changes were found and none of the two procedures were found to impair these parameters more than the other. However, a statistically significant drop in zinc element was noticed in SADI-S when compared to OAGB-MGB after 1 year of surgery (*p* = 0.004).

### Comorbidities

Of the 8 patients with T2D in the SADI-S group, T2D had resolved in 4 patients in 12 months, one patient had his medications gradually stepped down and stopped completely in 18 months. The other three patients had reduced their medication dosage. In the SADI-S group, abnormal A1C values decreased significantly.

Four patients in the SADI-S group had hypertension, two of them recovered over 1 year and became off medications. Seven patients had GERD symptoms before SADI-S, and symptoms had completely resolved over 1 year and medications were stopped in 3 patients. One patient had GERD symptoms controlled with PPI.

In patients who underwent OAGB-MGB, 6 patients had T2D and 5 had hypertension. In one patient, T2D and hypertension were completely resolved, while another 2 reported a decrease in their medications and improvement of their readings during 1-year follow-up.

### Complications

The number of complicated cases was 13 out of 49 for OAGB-MGB (27%) compared to 8 out of 42 for SADI-S (19%); *p* = 0.39.

Postoperative complications found in the OAGB-MGB group were anastomotic ulcer, bile reflux, de novo GERD, staple line leak, and revisional surgery, whereas steatorrhea, abdominal collection, and nutritional deficiency were observed in the SADI-S group.

Abdominal collection, the only early postoperative (< 30 days) complication following SADI-S, was treated conservatively.

Three patients in OAGB-MGB developed GERD postoperatively and another 3 patients had severe bile reflux which was confirmed by the HIDA scan. One of them was converted to Roux-en-Y gastric bypass (RYGB). Similarly, the procedure was converted to RYGB in a patient who developed a staple line leak shortly after OAGB-MGB. The 3 anastomotic ulcer cases were long-term complications and were treated medically with PPIs. The nutritional deficiency in both procedure groups needed hospitalization with total parenteral nutrition for 2 weeks. Even though steatorrhea was the main midterm (> 30 days and < 12 months) concern after SADI-S in 6 patients, no other revisional surgery was done.

Two patients with OAGB-MGB underwent another revisional surgery (which was SADI-S) due to weight regain.

No mortality was reported in the 2 study groups.

## Discussion

The present study is an effort to fill the gap in the literature by comparing two major bariatric revisional surgeries in patients with at least 1-year follow-up. The study demonstrates that both procedures have similar outcomes with regard to weight loss, remission of comorbidities, and nutritional deficiency; however, short- and long-term complications associated with SADI-S procedure were less when compared to OAGB-MGB. There is not much difference in the operating time between both procedures, reflecting the improvement of our learning curve [13].

Multiple studies had investigated the effectiveness of SADI-S, RYGB, or biliopancreatic diversion with DS in unsuccessful LSG [14–16]. It was generally agreed that the mechanism of failure should guide the selection of the second procedure. Dijkhorst et al. demonstrated that the conversion of LSG into SADI-S was associated with more weight loss; however, complications and nutritional deficiencies were similar [16]. One of the interesting findings in our study was the equivalent effect on weight loss for both SADI-S and OAGB-MGB after LSG. Similar results were observed in the mean BMI after 12 months for both groups (*p* = 0.46), even though the initial BMI for the SADI-S group was slightly higher.

Some of the previous studies involved mainly primary SADI-S, not as a revisional post-LSG, and follow-up was for a few years longer [14–16]. More distal alimentary anastomosis done in the ileum during SADI-S compared to biliopancreatic diversion was actually causing higher weight loss due to a potent ileal brake mediated by an enhanced secretion of the hormones peptide YY and glucagon-like peptide-1 which are associated with increased satiety and decreased food intake [7]. We could duplicate results of the previous studies in terms of weight loss and TWL% with a slight advantage of SADI-S over OAGB-MGB [7, 16–22]. Multiple studies reported EWL% as 70–80% with superiority for SADI procedure when compared to RYGB post-LSG [16, 20, 21]. These findings were in contrast to our results as there was no significant difference in EWL% between SADI-S and OAGB-MGB as revisional surgery (*p* > 0.05). Fulton et al. demonstrated that patients who underwent revisional surgeries experienced a significant decrease in BMI as evident in our study [22].

Our study demonstrated an equivalent positive effect on improving the comorbidities (T2D, hypertension, or lipid profile) over 1 year. Prior studies showed that SADI-S had a significant potential improvement of comorbidities when compared to other revisional surgeries [8, 23–25].

Sanchez-Pernaute et al. study reported a 52% remission rate of T2D over a 5-year follow-up, whereas Zaveri et al. reported an 81% remission rate over 4-year follow-up post-SADI-S [15, 26]. In our study, either remission or total resolution of T2D post-SADI-S or OAGB-MGB was 63% and 50% respectively. In addition, there was 50% in the SADI-S group and 60% in the OAGB-MGB group had either remission or total resolution of hypertension during 1-year follow-up. The impact on T2D and hypertension in our study was consistent with previous studies regardless of the revisional procedures [16, 27].

In addition, the SADI-S procedure showed more potential in controlling GERD progression over OAGB-MGB; 57% of patients who had GERD symptoms either stopped or decreased their PPI medications post-surgery in our study. This is consistent with some studies which identified SADI-S as a good revisional choice post-LSG for patients suffering from GERD symptoms [8, 28]. Our results showed that patient who underwent OAGB-MGB had more complications with a rate of 27% when compared to the 19% complication rate for patients underwent SADI-S procedure. Most of these complications were short term and reversible. The main OAGB-MGB postoperative complications were related to exacerbating the upper gastrointestinal symptoms and possibly creating GERD, anastomotic ulcer, and bile reflux.

Weight regain was another significant outcome after OAGB-MGB. Two patients (4%) regained weight within 1 year postoperatively in our study. Noun et al. study reported 13% weight regain after 18-months follow-up [29]. On the other hand, Lee et al. reported minimal weight regain in only 2% of patients [30].

Jamal et al. suggested that alterations in weight post-OAGB-MGB might be connected to the study group’s culture, eating habits, and genetic variability [31]. This weight regain could be reduced by diet modification, increasing the length of bypassed jejunum, or reducing the size of gastric pouch in intractable cases [30–32]. SADI-S procedure has been developed mainly to simplify the technique for biliopancreatic diversion (BPD) and many of the postoperative complications post-SADI-S procedure such as anastomotic leak or stricture were attributed to the learning curve and technical difficulties in the early phase of starting the SADI-S surgery as per previous studies [15, 33]. We did not encounter such complications in our patients postoperatively or during follow-up; however, one of our patients developed intra-abdominal collection postoperatively which was treated by aspiration and conservative treatment.

The leading complication in the SADI-S group was diarrhea/steatorrhea in 6 patients (14%); such complications were reported in various studies [33]. Although these complications were not severe as in patients who underwent BPD, it was common in the post-SADI-S procedure [33, 34]. In our study, this complication was actually temporary and resolved within 3 to 6 months in all patients. None of the patients in the OAGB-MGB group had a similar complication.

In our study, patients were found to experience a drop of vitamin D in the SADI-S group and anemia in the OAGB-MGB group which was also evident in previous studies [15, 16, 31, 33, 35, 36]. Creation of bypass anastomosis in itself causes malabsorption of fat-soluble vitamins due to poor mixing with bile salts, thus creating a further reduction in vitamin D [27].

SADI-S procedure results in higher vitamin B12 due to the increased availability of intrinsic factor (IF) when compared to other revisional surgeries since a larger portion of the stomach still exists to produce this glycoprotein [28]. However, a change in the level of vitamin B12 over 1 year for both procedures in our study was insignificant. In addition, the level of zinc was found to decrease more with SADI-S than OAGB-MGB in the present study (*p* = 0.004). Such deficiencies were not severe and patients were not symptomatic except for two patients (one in each group) who suffered from hypoalbuminemia and needed hospitalization with total parenteral nutrition for 2 weeks.

### Limitations

The retrospective design of the present study is one of the main limitations. Likewise, 1 year could be considered as a short-to-medium term follow-up and some of the patients lacked a regular follow-up. Therefore, further studies with long-term and regular follow-up are needed to compare the efficacy of both revisional procedures. Finally, diet and exercise habits of patients are crucial and might affect some of the lipid profile parameters; this was not addressed in our study. Additionally, most of the patients had their primary bariatric procedure (LSG) done outside our center. Nutritional complications after malabsorptive procedures require years of follow-up; therefore, a longer-term follow-up will be conducted including a comparison with other revisional procedures.

## Conclusions

SADI-S and OAGB-MGB are effective revisional procedures post unsuccessful LSG with comparable outcomes in terms of weight and BMI loss, remission of comorbidities, and nutritional deficiency in short-to-medium term follow-up. SADI-S procedure appears to cause less upper gastrointestinal complications and even looks a good option for patients suffering from GERD post-primary LSG. Moreover, the underlying bile reflux may get worse with OAGB-MGB. However, further prospective larger studies are needed.

## Electronic Supplementary Material

ESM 1 (DOC 87 kb)

## Data Availability

All data were given in the study analysis and tables.

## Abbreviations

BMI: Body mass index
LSG: Laparoscopic sleeve gastrectomy
SADI-S: Single anastomosis duodeno-ileostomy
OAGB-MGB: One anastomosis gastric bypass/mini gastric bypass
EWL%: Excess weight loss percentage
TWL%: Total weight loss percentage
GERD: Gastroesophageal reflux disease
T2D: Type 2 diabetes mellitus
A1C: Glycated hemoglobin A1C
